# Substance Use among Belgian Higher Education Students before and during the First Wave of the COVID-19 Pandemic

**DOI:** 10.3390/ijerph19074348

**Published:** 2022-04-05

**Authors:** Robert Tholen, Koen Ponnet, Guido Van Hal, Sara De Bruyn, Veerle Buffel, Sarah Van de Velde, Piet Bracke, Edwin Wouters

**Affiliations:** 1 Center for Population, Family and Health, University of Antwerp, 2000 Antwerp, Belgium; sara.debruyn@uantwerpen.be (S.D.B.); veerle.buffel@uantwerpen.be (V.B.); sarah.vandevelde@uantwerpen.be (S.V.d.V.); edwin.wouters@uantwerpen.be (E.W.); 2 Department of Communication Studies, imec-mict, Ghent University, 9000 Ghent, Belgium; koen.ponnet@ugent.be; 3 Department of Family Medicine and Population Health, Social Epidemiology and Health Policy, University of Antwerp, 2610 Antwerp, Belgium; guido.vanhal@uantwerpen.be; 4 Department of Sociology, Ghent University, 9000 Ghent, Belgium; piet.bracke@ugent.be

**Keywords:** substance use, higher education students, COVID-19, psychosocial distress, quarantine stressors

## Abstract

The COVID-19 pandemic prompted many countries to issue far-reaching policy measures that may have led to increased substance use. Higher education students may have been disproportionally affected due to the rearrangement of educational life and their susceptibility to psychosocial distress and substance use. The current study examined associations between pandemic-related stressors, psychosocial distress, and self-reported alcohol, tobacco, and cannabis use before and during the first wave of the pandemic. Data were collected in Belgium as part of the COVID-19 International Student Well-being Study (C19 ISWS) and analyzed using multinomial logistic regression analyses. The sample contained 18,346 higher education students aged 17 to 24 (75% women). Overall use of alcohol, tobacco, and cannabis as well as binge drinking decreased during the pandemic, perhaps due to limited social gatherings. Moving back to the parental home was associated with decreased substance use, while depressive symptoms were associated with increased substance use. Perceived threat and academic stress were associated with increased binge drinking among heavy bingers and increased tobacco use. Decreases among students who moved back to their parental home may be explained by increased informal social control. Increased substance use was associated with a number of stressors and psychosocial distress, which suggests that some students may have been self-medicating to manage their mental health amidst the pandemic. Public health policy concerning substance use may prove to be less effective if not tailored to particular subgroups within the student population.

## 1. Introduction

The COVID-19 (Coronavirus Disease 2019) pandemic started with a rapid spread of infections that led many countries to issue far-reaching policy measures. In Belgium where the current study was situated, the sharp rise in infections prompted the federal government to issue a stay-at-home order on 18 March 2020, which imposed strict social distancing measures, prohibited non-essential movements both within and outside of the country, and resulted in a long-term quarantine period [1]. Most restrictions installed during the first wave of the pandemic were lifted on 8 June 2020.

These measures may have had psychosocial repercussions. Earlier research identified a number of potential quarantine stressors including perceived threat, frustration, stigma, and financial losses [2]. One multi-country study found that perceived threat, stigma, and financial losses were associated with increased psychosocial distress [3]. Pandemic-related stressors and psychosocial distress have been found to be associated with increased use of alcohol [4,5,6,7], tobacco [6,7], and increased binge drinking [8]. 

Certain subpopulations such as higher education (HE) students may be especially vulnerable to this psychosocial impact—and its related substance use—as they were already displaying higher levels of psychosocial distress *pre*-COVID-19. Previous research has demonstrated that students were more likely to report psychosocial distress compared to older adults and non-student peers in the general population [9]. Stressors associated with student life included academic stress and dissatisfaction with the academic institution, changes in living situation, relationship problems, and lack of social support [9,10,11,12,13]. Moreover, studies have shown that alcohol use was more prominent among students compared to age-matched peers [14,15] and that attending college had increasingly become a risk factor for cannabis initiation [16]. In Flanders, one of three regions in Belgium, a last-year smoking prevalence of 30.7% was found among a representative sample of students in 2017 [17].

As a result of the COVID-19 pandemic, Belgian higher education institutions (HEIs) implemented major reorganizations such as switching to remote classes and cancellations or alterations to final examinations, internships, fieldwork, and student exchange programs [18]. Many students faced consequences in various aspects of their lives such as their living situation (e.g., moving back to the parental home), finances (e.g., loss of student job), social life (e.g., limited face-to-face meetings and no student activities), and emotional health problems (e.g., fear and anger) [19]. The pandemic may have further increased students’ susceptibility to psychosocial distress and increased substance use, underlining the need for further research. 

Previous research among HE students demonstrated that not relocating to live with parents [20], negative overall learning experience [21], academic stress, and academic dissatisfaction [22] were associated with psychosocial distress. Furthermore, psychosocial distress was found to be associated with increased use of alcohol [23,24], tobacco, and cannabis and increased binge drinking [23,25]. Academic stress was associated with increased tobacco use and binge drinking [25], while social support and social connectedness were associated with decreased alcohol use [26].

Studies testing several stressors and psychosocial distress factors simultaneously to uncover their associations with various types of substance use are limited. As such, there is a clear need for more comprehensive research examining associations between stressors, psychosocial distress, and substance use among students during the pandemic. Using data from the Belgian sample of the COVID-19 International Student Well-being Study (C19 ISWS), the following research question was addressed: to what extent are quarantine stressors and psychosocial distress associated with Belgian students’ self-reported substance use before and during the first wave of the COVID-19 pandemic?

## 2. Materials and Methods

### 2.1. Data Collection

Data stemmed from the C19 ISWS [27], a cross-sectional multi-country study performed across 26 countries and 133 HEIs, and were collected using a stratified convenience sampling design with an online questionnaire. The Belgian sample (*n* = 26,714, HEI = 13) was obtained between 26 April and 11 May 2020, during the first Belgian stay-at-home order (day 39 to day 54). The questionnaire was available in Dutch, French, and English. Translation of the core questionnaire was performed through a committee approach. Further information on the study procedures and questionnaire can be found in the study protocol [27,28]. Since this was a cross-sectional survey, self-reported behaviors before and during the pandemic were measured retrospectively.

### 2.2. Measures

The dependent variables in our study were (1) alcohol use; (2) binge drinking; (3) tobacco use; and (4) cannabis use. Students were asked to report on their behavior during two time intervals: before the COVID-19 outbreak (“the average situation during the month prior to the moment that the first COVID-19 measures (e.g., social distancing measures) were implemented”) and during the last week (“the week prior to filling out this survey”). Answers were categorized as decreased use, no change, or increased use. 

#### 2.2.1. Dependent Variables

Alcohol use—the number of glasses of alcohol (e.g., a glass of wine, a shot, or a glass of beer between 25 to 33 cl) on average per week before the COVID-19 outbreak and during the last week.

Tobacco use—the number of (e-)cigarettes and cigars vaped or smoked on average per day before the COVID-19 outbreak and during the last week.

Binge drinking—drinking six or more glasses of alcohol on a single occasion. The answer categories were: (1) (almost) never; (2) less than once a week; (3) once a week; (4) more than once a week; (5) (almost) daily; or (6) prefer not to say. Students who answered (1) (almost) never for both time intervals were treated as non-users.

Cannabis use—frequency of use of cannabis (marijuana, weed, or hash). The answer categories were the same as those for binge drinking, and answers were treated similarly.

#### 2.2.2. Psychosocial Distress

Depressive symptoms—the 8-item Center for Epidemiological Studies Depression Scale (CES-D8) [29], validated in previous research [30]. Answer categories ranged from 0 (none or almost none of the time) to 3 (all or almost all of the time). The scale had good reliability (α = 0.86).

#### 2.2.3. Quarantine Stressors

COVID-19 diagnosis—(un)confirmed COVID-19 diagnosis. The answer categories were: (1) Yes, confirmed by a lab test; (2) Yes, my health care provider told me I probably have it, but without a lab test; (3) I think I had or currently have COVID-19, but a health care provider did not confirm it; or (4) No, I do not think I had it or currently have it. Answers were categorized as (1) no or (2) (un)confirmed probably or yes.

Living situation—main living situation (excluding weekends and holidays) before the COVID-19 outbreak and during the last week. Answers were categorized as (1) parental home; (2) moved back to parental home; (3) outside of parental home; or (4) moved away from parental home.

Financial situation—the statement “I had sufficient financial resources to cover my monthly costs” was rated on a 5-point Likert scale (ranging from strongly agree to strongly disagree), for before the COVID-19 outbreak and during the last week. Answers were categorized as: (1) no change in financial situation; (2) increased financial situation; or (3) decreased financial situation.

Social activity—engaging in any of the nine listed social activities during the last week (e.g., “You talked to family and friends over the phone”). A count variable was created. Since social activity had a non-linear relationship with the logit of binge drinking, answers were categorized for these analyses (see Analytic Strategy).

Perceived threat—perceived threat was measured by a 6-item scale designed for this survey, with each item ranging from 0 (totally not) to 10 (very). Three items were worded slightly differently for students with a (supposed) COVID-19 diagnosis (e.g., “How worried are you to get (re-)infected with COVID-19?”). The scale had acceptable reliability (α = 0.79). 

Academic stress—academic stress was measured by a 4-item scale constructed for this survey, where each item was rated on a 5-point Likert scale ranging from 0 (strongly disagree) to 4 (strongly agree) (e.g., “I am concerned that I will not be able to successfully complete the academic year due to the COVID-19 outbreak.”). The scale showed acceptable reliability (α = 0.77).

Academic dissatisfaction—similar to academic stress, academic dissatisfaction was measured by a 4-item scale constructed for the survey, where each item was rated on a 5-point Likert scale (e.g., “The university/college provides a poorer quality of education during the COVID-19 outbreak than before.”). The scale showed moderate reliability (α = 0.68).

#### 2.2.4. Covariates

The covariates included were sex (0 = man; 1 = woman), age, parental educational attainment (0 = no HE degree among parents; 1 = HE degree among parents), study year (0 = first-year; 1 = higher year), type of HEI (0 = college of applied sciences; 1 = university), and having an underlying health condition that is a risk factor for COVID-19 (0 = no; 1 = yes). 

### 2.3. Analytic Strategy

To examine whether self-reported substance use differed significantly before and during the pandemic, we ran non-parametric dependent samples tests for continuous (Wilcoxon signed-rank tests) and categorical (Stuart-Maxwell tests) variables. Bivariate analyses were performed (see Supplementary 1, Tables S1–S4) followed by multinomial logistic regression analyses in which students who reported increased use, no change, and decreased use were distinguished. Adjusted odds ratios (AOR) and 95% confidence intervals (95% CI) were reported. The potential selectivity of the non-response and the robustness of the statistical analyses were assessed (see Supplementary 2), and the assumptions underlying multinomial logistic regression were tested. Social activity had a non-linear relationship with the logit of binge drinking; as such, the variable was categorized to distinguish between (1) median; (2) below median; and (3) above median (see Supplementary 3). To account for intensity of use, separate analyses were performed for light and heavy users, which can be found in Supplementary 4. Distinctions between light and heavy users were made based on previous research [31,32,33]. Finally, we repeated the analyses with students’ study field (based on ISCED [34]) included to examine the extent to which study field accounted for differences in self-reported changes in substance use. Results were similar across all analyses. All analyses were performed in IBM^®^ SPSS^®^ Version 28 (IBM Corp., Armonk, NY, USA).

## 3. Results

### 3.1. Sample Description

We included students who completed the survey (*n* = 21,270); were in the conventional student age range (17 to 24; *n* = 18,466); were not PhD students (*n* = 18,425); and identified as a man or woman (*n* = 18,346). Students who reported no substance use during both time intervals or had no valid answer on at least one time interval were treated as non-users and excluded from analyses. The final analyses included students with valid answers to all included questions (alcohol use: 13,404; binge drinking: 10,764; tobacco use: 2653; and cannabis use: 1804).

Table 1 shows the full information sample of 18,346 respondents. The majority of students were women (75%), higher year students (77%), and had at least one parent with a higher education degree (75%). More than half of the students lived with their parents both before and during the pandemic (i.e., continuously) (55%). Due to the pandemic, 32% moved back to their parental home. 

The self-reported overall use of all substances decreased significantly during the pandemic (all *p* < 0.001; alcohol use: Z = −58.74; binge drinking: χ^2^(4) = 82.21; tobacco use: Z = −4.57; and cannabis use: χ^2^(4) = 13.24). The weekly number of drinks decreased from a median of four (interquartile range (IQR) = 6) to a median of two (IQR = 4). The daily number of (e-)cigarettes and cigars decreased from a median of two (IQR = 6) to a median of one (IQR = 5). Binge drinking among students decreased drastically. While 41.1% (almost) never binge drank before COVID-19, 87.5% of students reported similar behavior during the pandemic. Of all students, 90.5% reported (almost) never using cannabis before the pandemic and 95.1% reported similar behavior during the pandemic. A marginal increase in (almost) daily users of cannabis was found from 1.0% (*n* = 188) to 1.5% (*n* = 268).

The prevalence rates for substance use were highest for alcohol (74.8%) followed by binge drinking (60.0%), tobacco (15.0%), and cannabis (10.1%) (Table 2). Self-reported decreases in use were less pronounced for tobacco (39.6%) and cannabis use (58.7%). 

### 3.2. Multinomial Logistic Regression Analyses

Table 3 shows the results for alcohol use and binge drinking. Analyses were repeated for light and heavy users (Tables S5 and S6). Students who moved back to their parental home were more likely to decrease their alcohol use than to not change their alcohol use compared to students who continuously lived with their parents (AOR: 1.80; 95% CI: 1.59–2.04). They were also more likely to decrease binge drinking than to not change their binge drinking frequency (AOR: 1.48; 95% CI: 1.23–1.78). Similar results were found among light users (Tables S5 and S6). Students living outside of the parental home continuously were less likely to decrease than to not change their binge drinking frequency (AOR: 0.61; 95% CI: 0.49–0.75). Reporting a decreased financial situation was associated with a higher likelihood of reporting increased alcohol use compared to reporting no changes (AOR: 1.29; 95% CI: 1.12–1.48) and a lower likelihood of reporting decreased binge drinking compared to reporting no changes (AOR: 0.75; 95% CI: 0.63–0.88). Students reporting more social activity were more likely to report decreased alcohol use rather than no changes (AOR: 1.07; 95% CI: 1.03–1.10). Heavy bingers who reported more perceived threat and academic stress were more likely to report increasing their binge drinking rather than no change (Table S6). No strong associations were found for academic dissatisfaction. Students reporting more depressive symptoms were more likely to report increased (AOR: 1.06; 95% CI: 1.05–1.08) or decreased (AOR: 1.04; 95% CI: 1.03–1.05) alcohol use as well as increased binge drinking (AOR: 1.08; 95% CI: 1.06–1.11) rather than no change. Similar results were found among both light and heavy users (Tables S5 and S6).

The results for tobacco use and cannabis use are presented in Table 4. Analyses were repeated for light and heavy users (Tables S7 and S8). Students who moved back to their parental home were less likely to increase smoking than to not change their smoking compared to students who continuously lived with their parents (AOR: 0.65; 95% CI: 0.50–0.83). Students continuously living outside of their parental home were more likely to report increased smoking (AOR: 1.69; 95% CI: 1.27–2.23) and less likely to report decreased smoking (AOR: 0.68; 95% CI: 0.52–0.91) compared to reporting no changes. Similar results were found among low-rate daily smokers (Table S7). Changes in financial situation were associated with a higher likelihood of reporting smoking less rather than reporting no changes. No significant associations were found for social activity and academic dissatisfaction. Students reporting more perceived threat (AOR: 1.10; 95% CI: 1.03–1.17), more academic stress (AOR: 1.25; 95% CI: 1.07–1.45), and more depressive symptoms (AOR: 1.06; 95% CI: 1.03–1.08) were more likely to report increased smoking rather than no changes. Similar results were found for academic stress among low-rate daily smokers and for depressive symptoms among both low-rate and high-rate daily smokers (Table S7).

Students who moved back to their parental home were more likely to decrease their cannabis use than to not change their use (AOR: 1.83; 95% CI: 1.32–2.53) compared to students who continuously lived with their parents. Similar results were found among both light and heavy cannabis users (Table S8). Students who lived outside of their parental home (AOR: 0.40; 95% CI: 0.28–0.56) or who moved away from their parents (AOR: 0.26; 95% CI: 0.12–0.57) were less likely to report decreased use of cannabis rather than no changes. Students with increased finances were less likely to report increased cannabis use (AOR: 0.45; 95% CI: 0.24–0.83). Similar results were found among heavy cannabis users (Table S8). No strong associations were found for social activity, perceived threat, academic stress, and academic dissatisfaction. Reporting more depressive symptoms was associated with reporting increased cannabis use (AOR: 1.06; 95% CI: 1.03–1.10) compared to reporting no changes. Similar results were found among heavy cannabis users (Table S8).

## 4. Discussion

Overall, the self-reported use of all four substances significantly decreased during the pandemic. Prevalence rates of 74.8% for alcohol, 60.0% for binge drinking, 15.0% for tobacco, and 10.1% for cannabis were observed. Compared to a German study based on the C19 ISWS data, prevalence rates for alcohol and binge drinking were higher in our sample, while rates for tobacco and cannabis use were lower. No changes in tobacco and cannabis use and decreased binge drinking were reported [23]. A French study using the C19 ISWS data observed significant decreases in the prevalence of tobacco use, binge drinking, and cannabis use. Prevalence rates for tobacco use were comparable but rates for binge drinking and cannabis use were lower [25]. In a Dutch study based on the C19 ISWS data [35], prevalence rates for weekly binge drinking before the pandemic and smoking were comparable and rates for cannabis were higher. Additionally, a higher proportion of students reported weekly binge drinking during the pandemic (13.9% vs. 5.3%).

The differences in results may in part be explained by the use of different categorizations (e.g., distinguishing between occasional and regular users), applying a different or no age range, and importantly, by the inclusion of non-users in the analyses. Non-users were excluded in the current study to decrease the heterogeneity of the no-change group.

The overall use of substances may have decreased as a result of more limited opportunities to attend private and public social gatherings [36]. Research has shown that students mostly drink during social occasions and for social and enhancement motives [37,38]. Similarly, since students have been found to often smoke a relatively low number of cigarettes per day [17], the number of social smokers among students may be relatively high, and the overall decrease of tobacco use may be attributed to this group. Cannabis use may have decreased due to a reduced availability and travel restrictions, as the most common way for Belgian students to obtain cannabis is through cannabis dispensaries in the Netherlands. Contacting local dealers may have become more complicated, and prices may have increased due to a lower stock [39].

Decreases in alcohol use, binge drinking, and cannabis use were more likely to be reported by students moving back to their parental home. Increases in tobacco use were less likely to be reported by these students. Students living outside of the parental home continuously were less likely to report decreased binge drinking, tobacco use, and cannabis use and more likely to report increased tobacco use. Living away from parental control in environments with more permissive social norms has been demonstrated to be associated with increased substance use [38,40,41]. The increased informal social control may thus have led to decreased use among students moving back in with their parents. 

Perceived threat and academic stress were found to be associated with a higher likelihood of reporting increased binge drinking among heavy bingers and with a higher likelihood of reporting increased tobacco use. Depressive symptoms were associated with a higher likelihood of reporting increased use among all types of substance use and among both light and heavy users (except for light cannabis users). Earlier research found associations between academic stress, increased binge drinking, and increased tobacco use [25]. Associations were also found between depressive symptoms and increased alcohol [23,42], tobacco, and cannabis use [23,25], as well as increased binge drinking [25]. Moreover, studies have demonstrated associations between stressors identified in this study and psychosocial distress: perceived threat [22], not moving back to the parental home [20], living alone compared to living with other people [22], and academic stress [22]. It thus appears that some students may have been self-medicating [43] during the first stay-at-home order to manage their psychosocial distress amidst a period of instability and containment measures. However, since the current study made use of cross-sectional data, no causal inferences can be made. More longitudinal research is required to further understand the mechanisms at play.

### 4.1. Strengths and Limitations

The current study examined several COVID-19 stressors and psychosocial distress simultaneously in a large sample of HE students. The results of this study should be considered with a few limitations in mind. The data were assembled by means of stratified convenience sampling and were cross-sectional in nature, meaning that the study population was not representative of Belgian HE students and only questioned once. Response rates were unavailable, as several HEIs implemented alternative recruitment methods besides e-mail distribution such as the use of newsletters, student-specific platforms, and social media platforms [27]. In the survey, students were asked to report on their substance use, both currently and retrospectively. It may be that objective longitudinal measurements lead to different results. The data may suffer from nonresponse bias, which may have led to under- or overestimation of associations. Furthermore, the data reported here were obtained during the first wave of the pandemic. Initial studies on students’ substance use during subsequent waves reported both increases and decreases in (problematic) alcohol use [44,45]. As such, it remains unclear how the results presented here can be extrapolated to subsequent waves of the pandemic.

Nevertheless, the current study provides important insights into how self-reported changes in substance use behaviors are associated with quarantine stressors and psychosocial distress. Lessons learned from our study may be helpful for understanding how people may cope with potential future global (health) crises.

### 4.2. Future Research and Implications

The current study provides insight into the extent to which quarantine stressors and psychosocial distress were associated with Belgian students’ self-reported substance use. Although potential negative ramifications were addressed, we found that students overall were less likely to report using substances during the pandemic. More in-depth research is necessary to uncover why students who reported changing their behavior reacted differently to quarantine stressors. On a psychological level, this may be due to different coping strategies being used to deal with the profound changes that occurred as a result of the COVID-19 pandemic.

Considering that our study reported on behaviors during the first wave of the pandemic, it is important to analyze data obtained during subsequent waves. In particular, the finding that increased substance use was associated with quarantine stressors and psychosocial distress is alarming, and future studies should examine whether these associations persist over time. 

The findings presented here are in line with two meta-analyses on alcohol use that report an overall decrease of use during COVID-19, while increased use was found to be associated with several types of stressors and psychosocial distress [46,47]. Future studies should investigate whether these trends apply to the use of tobacco and cannabis as well, as is suggested by the results of the current study.

From the societal perspective, it is important to uncover the extent to which policy measures initiated by HEIs and governments were associated with substance use. Such cross-country comparisons would enhance our understanding of how broader social contexts contribute to associations between COVID-19 stressors, psychosocial distress, and substance use. They can also aid policy makers in deciding which policy measures would be most effective, and whether such measures should be tailored to particular subgroups of the student population.

## 5. Conclusions

The current study is among the first to examine several quarantine stressors and psychosocial distress simultaneously in a large sample of higher education students. During the first wave of the COVID-19 pandemic, overall substance use among Belgian higher education students decreased. Increases in the use of substances were associated with a number of stressors and psychosocial distress. As such, public health policy concerning substance use may prove to be less effective if not tailored to particular subgroups within the student population. The results of this study may inform further cross-country research aimed at enlarging our understanding of how broader social contexts contribute to associations between quarantine stressors, psychosocial distress, and changes in substance use. 

## Figures and Tables

**Table 1:** Full information sample with percentage distributions (%) for categorical variables, and median ($\bar{X}$) and Tukey’s hinges (25th percentile (Q_1_) and 75th percentile (Q_3_)) for continuous non-normally distributed variables (*n* = 18,346).

			%	*n*	$\bar{X}$	Q_1_	Q_3_
**Covariates**	Sex	Men	25.3	4637			
		Women	74.7	13,709			
	Age (17–24)				20	19	22
	Parent(s) HE degree	No	25.5	4615			
		Yes	74.5	13,509			
		Missing	1.2	222			
	Study year	First-year	22.9	4201			
		Higher year	77.1	14,145			
	Institution type	College of applied sciences	39.5	7203			
		University	60.5	11,055			
		Missing	0.5	88			
	Health condition	No	89.4	16,206			
		Yes	10.6	1921			
		Missing	1.2	219			
	Study field	Education	6.2	1134			
		Humanities and arts	10.6	1950			
		Social sciences, business and law	33.3	6113			
		Science	7.9	1450			
		Engineering, manufacturing and construction	11.3	2073			
		Agriculture	2.7	487			
		Health and welfare	22.2	4075			
		Services	1.5	270			
		Other	0.1	25			
		Missing	4.2	769			
**Stressors**	COVID-19 diagnosis	No	88.5	16,237			
		Yes	11.5	2103			
		Missing	0.0	6			
	Living situation	Parental home	54.9	10,065			
		Moved back to parental home	31.8	5836			
		Outside of parental home	11.2	2054			
		Moved away from parental home	2.1	391			
	Financial situation	Decrease	27.4	5023			
		No change	67.3	12,356			
		Increase	5.3	967			
	Social activity (0–9)				5	3	6
	Perceived threat (0–10)				5.67	4.50	6.83
	Academic stress (0–4)				3.00	2.50	3.75
	Academic dissatisfaction (0–4)				2.00	1.50	2.50
**Psychosocial** **distress**	Depressive symptoms (0–24)				10	7	14
**Substance use**	Number of drinks weekly	Before COVID-19			4	2	8
		During COVID-19			2	0	4
		Non-drinkers		4630			
	Number of (e-)cigarettes/cigars daily	Before COVID-19			2	0	6
		During COVID-19			1	0	5
		Non-smokers		15,508			
		Missing		102			
	Binge drinking before COVID-19	(Almost) never	41.1	7542			
		Less than once a week	31.1	5700			
		Once a week	19.1	3507			
		More than once a week	8.1	1492			
		(Almost) daily	0.5	86			
		Missing	0.1	19			
	Binge drinking during COVID-19	(Almost) never	87.5	16,047			
		Less than once a week	7.2	1328			
		Once a week	3.3	599			
		More than once a week	1.7	303			
		(Almost) daily	0.3	59			
		Missing	0.1	10			
	Non-bingers total			7335			
	Cannabis use before COVID-19	(Almost) never	90.5	16,609			
		Less than once a week	5.5	1,013			
		Once a week	1.4	251			
		More than once a week	1.3	244			
		(Almost) daily	1.0	188			
		Missing	0.2	41			
	Cannabis use during COVID-19	(Almost) never	95.1	17,441			
		Less than once a week	1.4	266			
		Once a week	0.9	156			
		More than once a week	1.0	183			
		(Almost) daily	1.5	268			
		Missing	0.2	32			
	Non-cannabis users total			16,451			

**Table 2:** Self-reported substance use behaviors for students with valid answers to all variables included in final models.

Dependent Variable	Decrease	No Change	Increase	Valid N	Prevalence ^1^
Alcohol use	64.6%	12.7%	22.7%	13,404	74.8%
Binge drinking	87.4%	6.8%	5.8%	10,764	60.0%
Tobacco use	39.6%	32.5%	27.9%	2653	15.0%
Cannabis use	58.7%	18.5%	22.8%	1804	10.1%

^1^ Prevalence rates based on full information sample (minus missing values).

**Table 3:** Multinomial regression analyses for alcohol use and binge drinking (*n* = 13,404; *n* = 10,764).

		Alcohol Use				Binge Drinking			
		Increase vs. No Change		Decrease vs. No Change		Increase vs. No Change		Decrease vs. No Change	
		AOR	95% CI	AOR	95% CI	AOR	95% CI	AOR	95% CI
Sex	Men	1.00		1.00		1.00		1.00	
	Women	0.87	0.75–1.01	0.71 ***	0.62–0.81	0.85	0.67–1.09	1.12	0.94–1.33
Age (17–24)		1.00	0.96–1.04	0.82 ***	0.79–0.85	1.11 **	1.03–1.20	0.84 ***	0.79–0.89
Parent(s) HE degree	No	1.00		1.00		1.00		1.00	
	Yes	1.02	0.88–1.17	1.02	0.89–1.15	1.11	0.86–1.42	1.09	0.92–1.31
Study year	First-year	1.00		1.00		1.00		1.00	
	Higher year	1.00	0.82–1.20	1.09	0.91–1.28	0.79	0.57–1.10	1.35 *	1.07–1.70
Institution type	College of applied sciences	1.00		1.00		1.00		1.00	
	University	0.90	0.79–1.02	1.32 ***	1.18–1.48	0.86	0.68–1.08	1.38 ***	1.17–1.62
Health condition	No	1.00		1.00		1.00		1.00	
	Underlying condition	1.33 **	1.08–1.62	1.12	0.93–1.35	1.07	0.78–1.48	0.81	0.64–1.03
COVID-19 diagnosis	No	1.00		1.00		1.00		1.00	
	(Un)confirmed probably or yes	0.95	0.79–1.14	1.01	0.85–1.19	1.03	0.75–1.42	1.04	0.83–1.32
Living situation	Parental home	1.00		1.00		1.00		1.00	
	Moved back to parental home	0.95	0.82–1.09	1.80 ***	1.59–2.04	0.98	0.75–1.28	1.48 ***	1.23–1.78
	Outside of parental home	1.01	0.84–1.21	1.00	0.84–1.18	1.22	0.91–1.64	0.61 ***	0.49–0.75
	Moved away from parental home	1.72 **	1.18–2.50	0.99	0.69–1.43	1.99 *	1.17–3.38	0.64 *	0.41–0.98
Financial situation	No change	1.00		1.00		1.00		1.00	
	Decrease	1.29 ***	1.12–1.48	1.11	0.98–1.26	1.13	0.90–1.43	0.75 ***	0.63–0.88
	Increase	1.16	0.87–1.55	1.41 **	1.09–1.81	1.38	0.85–2.23	1.08	0.76–1.53
Social activity (0–9)		1.01	0.97–1.05	1.07 ***	1.03–1.10				
Social activity	Median					1.00		1.00	
	Below median					1.15	0.87–1.54	1.09	0.89–1.33
	Above median					1.04	0.77–1.40	0.91	0.75–1.12
Perceived threat (0–10)		1.01	0.97–1.04	0.97 *	0.93–1.00	1.03	0.97–1.10	1.00	0.96–1.05
Academic stress (0–4)		1.03	0.94–1.12	0.92 *	0.85–0.99	1.21 *	1.02–1.43	0.94	0.84–1.05
Academic dissatisfaction (0–4)		1.05	0.96–1.15	1.11 *	1.02–1.20	1.06	0.90–1.24	0.99	0.88–1.11
Depressive symptoms (0–24)		1.06 ***	1.05–1.08	1.04 ***	1.03–1.05	1.08 ***	1.06–1.11	1.01	0.99–1.03

*** *p* < 0.001; ** *p* < 0.01; * *p* < 0.05.

**Table 4:** Multinomial regression analyses for tobacco use and cannabis use (*n* = 2653; *n* = 1804).

		Tobacco Use				Cannabis Use			
		Increase vs. No Change		Decrease vs. No Change		Increase vs. No Change		Decrease vs. No Change	
		AOR	95% CI	AOR	95% CI	AOR	95% CI	AOR	95% CI
Sex	Men	1.00		1.00		1.00		1.00	
	Women	0.88	0.70–1.11	1.13	0.92–1.39	1.23	0.89–1.69	1.20	0.91–1.57
Age (17–24)		1.17 ***	1.08–1.26	1.08 *	1.01–1.15	1.00	0.90–1.11	0.87 **	0.79–0.95
Parent(s) HE degree	No	1.00		1.00		1.00		1.00	
	Yes	0.64 ***	0.50–0.82	1.05	0.83–1.33	0.84	0.59–1.19	1.00	0.73–1.37
Study year	First-year	1.00		1.00		1.00		1.00	
	Higher year	0.69 *	0.51–0.92	0.88	0.68–1.15	1.10	0.72–1.69	1.38	0.96–2.00
Institution type	College of applied sciences	1.00		1.00		1.00		1.00	
	University	0.61 ***	0.49–0.76	0.66 ***	0.54–0.80	1.21	0.89–1.65	1.70 ***	1.30–2.22
Health condition	No	1.00		1.00		1.00		1.00	
	Underlying condition	1.27	0.92–1.74	0.89	0.65–1.20	0.91	0.56–1.47	1.08	0.71–1.63
COVID-19 diagnosis	No	1.00		1.00		1.00		1.00	
	(Un)confirmed probably or yes	1.38 *	1.03–1.85	1.12	0.85–1.47	1.13	0.75–1.69	1.05	0.73–1.51
Living situation	Parental home	1.00		1.00		1.00		1.00	
	Moved back to parental home	0.65 ***	0.50–0.83	1.07	0.87–1.32	1.05	0.70–1.56	1.83 ***	1.32–2.53
	Outside of parental home	1.69 ***	1.27–2.23	0.68 **	0.52–0.91	1.13	0.78–1.64	0.40 ***	0.28–0.56
	Moved away from parental home	1.00	0.56–1.79	0.73	0.42–1.26	1.29	0.63–2.64	0.26 ***	0.12–0.57
Financial situation	No change	1.00		1.00		1.00		1.00	
	Decrease	1.41 **	1.12–1.77	1.58 ***	1.28–1.94	1.19	0.87–1.64	0.90	0.68–1.21
	Increase	1.11	0.72–1.72	2.10 ***	1.48–2.98	0.45 *	0.24–0.83	0.95	0.61–1.47
Social activity (0–9)		1.02	0.96–1.09	1.00	0.94–1.05	1.07	0.98–1.17	0.95	0.88–1.03
Perceived threat (0–10)		1.10 **	1.03–1.17	1.07 *	1.01–1.13	1.04	0.95–1.14	0.93	0.86–1.01
Academic stress (0–4)		1.25 **	1.07–1.45	1.07	0.94–1.22	0.98	0.79–1.22	0.92	0.76–1.11
Academic dissatisfaction (0–4)		0.97	0.83–1.12	1.01	0.88–1.16	1.06	0.85–1.31	1.02	0.85–1.24
Depressive symptoms (0–24)		1.06 ***	1.03–1.08	1.00	0.98–1.02	1.06 ***	1.03–1.10	1.20	0.91–1.57

*** *p* < 0.001; ** *p* < 0.01; * *p* < 0.05.

## Data Availability

The rough data and analytical sample underlying this article are available in the public domain: https://zenodo.org/record/5946964 (accessed on 1 March 2022).

## Supplementary Materials

The following supporting information can be downloaded at: https://www.mdpi.com/article/10.3390/ijerph19074348/s1, **Supplementary 1.** Bivariate analyses. Table S1. Bivariate analyses for alcohol use (*n* = 13,404); Table S2. Bivariate analyses for binge drinking (*n* = 10,764); Table S3. Bivariate analyses for tobacco use (*n* = 2653); Table S4. Bivariate analyses for cannabis use (*n* = 1804). **Supplementary 2.** Missing value and sensitivity analyses. **Supplementary 3.** Assumption testing. **Supplementary 4.** Control analyses. Table S5. Multinomial regression analyses for alcohol use: light drinkers (*n* = 11,508) and heavy drinkers (*n* = 1896); Table S6. Multinomial regression analyses for binge drinking: light bingers (*n* = 9826) and heavy bingers (*n* = 938); Table S7. Multinomial regression analyses for tobacco use: low-rate daily smokers (*n* = 2038) and high-rate daily smokers (*n* = 615); Table S8. Multinomial regression analyses for cannabis use: light cannabis users (*n* = 1212) and heavy cannabis users (*n* = 592).
