# Supplementary material for: Substance Use among Belgian Higher Education Students before and during the First Wave of the COVID-19 Pandemic
Source: Int J Environ Res Public Health. 2022 Apr 5;19(7):4348. doi: 10.3390/ijerph19074348 (PMC8998911; doi:10.3390/ijerph19074348)
Supplement: Supplementary file 1 [file ijerph-19-04348-s001.zip › Supplementary_1_bivariate_analyses.pdf]

International Journal of
Environmental Research and Public Health

MDPI

Supplementary 1

Bivariate analyses

Table S1. Bivariate analyses for alcohol use (N=13,404)

Variable	Values	Increase N=3,043	No change N=1,696	Decrease N=8,665	Chi-square	Effect size
Sex	Male	23%	22%	28%	$\chi^2(2) = 41.262$***	$\phi_c = 0.055$
	Female	77%	78%	72%		
Age[1]	17-24	21 (20 ; 22)	21 (20 ; 22)	20 (19 ; 22)	$\chi^2(2) = 261.471$***	$\eta^2 = 0.018$
Parent(s) HE degree	No	26%	24%	21%	$\chi^2(2) = 25.727$***	$\phi_c = 0.044$
	Yes	74%	76%	79%		
Study year	First-year	18%	17%	23%	$\chi^2(2) = 52.084$***	$\phi_c = 0.062$
	Higher year	82%	83%	77%		
Institution type	College of applied sciences	44%	41%	35%	$\chi^2(2) = 84.867$***	$\phi_c = 0.080$
	University	56%	59%	65%		
Health condition	No	88%	91%	90%	$\chi^2(2) = 21.683$***	$\phi_c = 0.040$
	Underlying condition	12%	9%	10%		
COVID-19 diagnosis	No	88%	88%	88%	$\chi^2(2) = 0.384$, $p = 0.825$	$\phi_c = 0.005$
	(Un)confirmed probably or yes	12%	12%	12%		
Living situation	Parental home	56%	56%	47%	$\chi^2(6) = 337.665$***	$\phi_c = 0.112$
	Moved back to parental home	25%	27%	41%		
	Outside of parental home	15%	15%	10%		
	Moved away from parental home	4%	2%	2%		
Financial situation	Decrease	34%	26%	26%	$\chi^2(4) = 77.952$***	$\phi_c = 0.054$
	No change	61%	70%	68%		
	Increase	5%	4%	6%		
Social activity[1]	0-9	5 (3 ; 6)	5 (4 ; 6)	5 (4 ; 6)	$\chi^2(2) = 18.375$***	$\eta^2 = 0.003$
Perceived threat[1]	0-10	5.83 (4.67 ; 7.00)	5.67 (4.50 ; 6.83)	5.60 (4.50 ; 6.67)	$\chi^2(2) = 54.919$***	$\eta^2 = 0.011$
Academic stress[1]	0-4	3.00 (2.50 ; 3.75)	3.00 (2.25 ; 3.50)	3.00 (2.25 ; 3.50)	$\chi^2(2) = 46.212$***	$\eta^2 = 0.003$
Academic dissatisfaction[1]	0-4	2.00 (1.50 ; 2.50)	2.00 (1.25 ; 2.25)	2.00 (1.50 ; 2.50)	$\chi^2(2) = 23.793$***	$\eta^2 = 0.001$
Depressive symptoms[1]	0-24	11 (7 ; 15)	9 (6 ; 13)	10 (7 ; 14)	$\chi^2(2) = 107.981$***	$\eta^2 = 0.004$

*** $p < .001$; ** $p < .01$; * $p < .05$; ϕ_c = Cramer's V; η^2 = Eta squared

[1] Median, 25th percentile and 75th percentile (Tukey's hinges)

Table S2. Bivariate analyses for binge drinking (N=10,764)

Variable	Values	Increase N=621	No change N=738	Decrease N=9,405	Chi-square	Effect size
Sex	Male	30%	30%	28%	$\chi^2(2) = 2.139$,	$\phi_c = 0.014$
	Female	70%	70%	72%	$p = 0.343$	
Age[1]	17-24	21 (20 ; 23)	21 (20 ; 22)	20 (19 ; 22)	$\chi^2(2) = 114.811$***	$\eta^2 = 0.011$
Parent(s) HE degree	No	27%	26%	22%	$\chi^2(2) = 16.115$***	$\phi_c = 0.039$
	Yes	73%	74%	78%		
Study year	First-year	21%	20%	22%	χ2(2) = 2.131,	$\phi_c = 0.014$
	Higher year	79%	80%	78%	$p = 0.345$	
Institution type	College of applied sciences	46%	43%	36%	$\chi^2(2) = 40.076$***	$\phi_c = 0.061$
	University	54%	57%	64%		
Health condition	No	85%	88%	90%	$\chi^2(2) = 19.719$***	$\phi_c = 0.043$
	Underlying condition	15%	12%	10%		
COVID-19 diagnosis	No	86%	88%	88%	$\chi^2(2) = 2.457$,	$\phi_c = 0.015$
	(Un)confirmed probably or yes	14%	12%	12%	$p = 0.293$	
Living situation	Parental home	47%	51%	50%	$\chi^2(6) = 265.028$***	$\phi_c = 0.111$
	Moved back to parental home	23%	25%	38%		
	Outside of parental home	24%	20%	10%		
	Moved away from parental home	6%	4%	2%		
Financial situation	Decrease	44%	36%	27%	$\chi^2(4) = 113.151$***	$\phi_c = 0.072$
	No change	50%	59%	67%		
	Increase	6%	5%	6%		
Social activity	Below median	46%	40%	44%	$\chi^2(4) = 7.897$, $p = 0.095$	$\phi_c = 0.019$
	Median	20%	23%	23%		
	Above median	34%	37%	33%		
Perceived threat[1]	0-10	5.83 (4.67 ; 7.17)	5.50 (4.50 ; 6.83)	5.67 (4.50 ; 6.67)	$\chi^2(2) = 18.514$***	$\eta^2 = 0.002$
Academic stress[1]	0-4	3.50 (2.75 ; 4.00)	3.00 (2.50 ; 3.50)	3.00 (2.50 ; 3.50)	$\chi^2(2) = 71.094$***	$\eta^2 = 0.006$
Academic dissatisfaction[1]	0-4	2.00 (1.50 ; 2.75)	2.00 (1.50 ; 2.50)	2.00 (1.50 ; 2.50)	$\chi^2(2) = 34.193$***	$\eta^2 = 0.004$
Depressive symptoms[1]	0-24	13 (9 ; 17)	10 (7 ; 14)	10 (7 ; 14)	$\chi^2(2) = 108.649$***	$\eta^2 = 0.012$

*** $p < .001$; ** $p < .01$; * $p < .05$; ϕ_c = Cramer's V; η^2 = Eta squared

[1] Median, 25th percentile and 75th percentile (Tukey's hinges)

Table S3. Bivariate analyses for tobacco use (N=2,653)

Variable	Values	Increase N=739	No change N=864	Decrease N=1,050	Chi-square	Effect size
Sex	Male	34%	37%	32%	$\chi^2(2) = 5.043$, $p = 0.080$	$\phi_c = 0.044$
	Female	66%	63%	68%		
Age[1]	17-24	21 (20 ; 23)	20 (19 ; 22)	21 (19 ; 22)	$\chi^2(2) = 20.147$***	$\eta^2 = 0.008$
Parent(s) HE degree	No	32%	19%	21%	$\chi^2(2) = 47.257$***	$\phi_c = 0.133$
	Yes	68%	81%	79%		
Study year	First-year	24%	23%	23%	$\chi^2(2) = 0.097$, $p = 0.953$	$\phi_c = 0.006$
	Higher year	76%	77%	77%		
Institution type	College of applied sciences	45%	32%	42%	$\chi^2(2) = 33.544$***	$\phi_c = 0.112$
	University	55%	68%	58%		
Health condition	No	85%	90%	90%	$\chi^2(2) = 12.597$**	$\phi_c = 0.069$
	Underlying condition	15%	10%	10%		
COVID-19 diagnosis	No	82%	88%	86%	$\chi^2(2) = 10.757$**	$\phi_c = 0.064$
	(Un)confirmed probably or yes	18%	12%	14%		
Living situation	Parental home	44%	42%	44%	$\chi^2(6) = 112.901$***	$\phi_c = 0.146$
	Moved back to parental home	22%	37%	40%		
	Outside of parental home	31%	18%	13%		
	Moved away from parental home	3%	3%	3%		
Financial situation	Decrease	45%	29%	38%	$\chi^2(4) = 66.208$***	$\phi_c = 0.112$
	No change	49%	65%	52%		
	Increase	6%	6%	10%		
Social activity[1]	0-9	5 (4 ; 6)	5 (4 ; 6)	5 (4 ; 6)	$\chi^2(2) = 0.153$, $p = 0.926$	$\eta^2 = 0.000$
Perceived threat[1]	0-10	5.83 (4.67 ; 7.17)	5.33 (4.00 ; 6.67)	5.67 (4.50 ; 7)	$\chi^2(2) = 35.011$***	$\eta^2 = 0.013$
Academic stress[1]	0-4	3.25 (2.75 ; 3.75)	3.00 (2.25 ; 3.50)	3.25 (2.50 ; 3.75)	$\chi^2(2) = 49.380$***	$\eta^2 = 0.016$
Academic dissatisfaction[1]	0-4	2.25 (1.50 ; 2.75)	2.00 (1.50 ; 2.50)	2.00 (1.50 ; 2.50)	$\chi^2(2) = 15.784$***	$\eta^2 = 0.005$
Depressive symptoms[1]	0-24	13 (9 ; 17)	11 (7 ; 15)	11 (8 ; 15)	$\chi^2(2) = 61.770$***	$\eta^2 = 0.024$

*** $p < .001$; ** $p < .01$; * $p < .05$; ϕ_c = Cramer's V; η^2 = Eta squared

[1] Median, 25th percentile and 75th percentile (Tukey's hinges)

Table S4. Bivariate analyses for cannabis use (N=1,804)

Variable	Values	Increase N=411	No change N=334	Decrease N=1,059	Chi-square	Effect size
Sex	Male	35%	44%	42%	$\chi^2(2) = 8.100^*$	$\phi_c = 0.067$
	Female	65%	56%	58%		
Age[1]	17-24	21 (20 ; 22.50)	21 (20 ; 22)	20 (19 ; 22)	$\chi^2(2) = 37.161^{***}$	$\eta^2 = 0.021$
Parent(s) HE degree	No	28%	24%	21%	$\chi^2(2) = 7.768^*$	$\phi_c = 0.066$
	Yes	72%	76%	79%		
Study year	First-year	21%	23%	24%	$\chi^2(2) = 2.372$, $p = 0.305$	$\phi_c = 0.036$
	Higher year	79%	77%	76%		
Institution type	College of applied sciences	41%	45%	34%	$\chi^2(2) = 14.089^{***}$	$\phi_c = 0.088$
	University	59%	55%	66%		
Health condition	No	89%	89%	89%	$\chi^2(2) = 0.008$, $p = 0.996$	$\phi_c = 0.002$
	Underlying condition	11%	11%	11%		
COVID-19 diagnosis	No	83%	85%	85%	$\chi^2(2) = 1.164$, $p = 0.559$	$\phi_c = 0.025$
	(Un)confirmed probably or yes	17%	15%	15%		
Living situation	Parental home	36%	40%	40%	$\chi^2(6) = 176.163^{***}$	$\phi_c = 0.221$
	Moved back to parental home	22%	22%	45%		
	Outside of parental home	37%	34%	14%		
	Moved away from parental home	5%	5%	1%		
Financial situation	Decrease	45%	35%	30%	$\chi^2(4) = 39.551^{***}$	$\phi_c = 0.105$
	No change	51%	55%	60%		
	Increase	4%	10%	10%		
Social activity[1]	0-9	5 (4 ; 6)	5 (4 ; 6)	5 (4 ; 6)	$\chi^2(2) = 4.793$, $p = 0.091$	$\eta^2 = 0.003$
Perceived threat[1]	0-10	5.50 (4.50 ; 7.00)	5.42 (4.00 ; 6.50)	5.17 (4.00 ; 6.33)	$\chi^2(2) = 18.739^{***}$	$\eta^2 = 0.011$
Academic stress[1]	0-4	3.25 (2.50 ; 3.75)	3.00 (2.50 ; 3.75)	3.00 (2.50 ; 3.75)	$\chi^2(2) = 7.707^*$	$\eta^2 = 0.004$
Academic dissatisfaction[1]	0-4	2.00 (1.50 ; 2.75)	2.00 (1.50 ; 2.50)	2.00 (1.50 ; 2.50)	$\chi^2(2) = 1.916$, $p = 0.384$	$\eta^2 = 0.001$
Depressive symptoms[1]	0-24	13 (9 ; 17)	11 (7 ; 15)	12 (8 ; 15)	$\chi^2(2) = 20.411^{***}$	$\eta^2 = 0.012$

*** $p < .001$; ** $p < .01$; * $p < .05$; ϕ_c = Cramer's V; η^2 = Eta squared

[1] Median, 25th percentile and 75th percentile (Tukey's hinges)
