# Supplementary material for: Substance Use among Belgian Higher Education Students before and during the First Wave of the COVID-19 Pandemic
Source: Int J Environ Res Public Health. 2022 Apr 5;19(7):4348. doi: 10.3390/ijerph19074348 (PMC8998911; doi:10.3390/ijerph19074348)
Supplement: Supplementary file 1 [file ijerph-19-04348-s001.zip › Supplementary_2_missing_sensitivity_analyses.pdf]

International Journal of
Environmental Research and Public Health

MDPI

Supplementary 2

Missing value and sensitivity analyses

Missing value analyses

Little's MCAR (missing completely at random) tests demonstrated that the data were not MCAR for all dependent variables (alcohol use: $\chi^2(64) = 212.851$, $p < .001$; binge drinking: $\chi^2(49) = 160.395$, $p < .001$; tobacco use: $\chi^2(54) = 86.629$, $p = .003$; cannabis use: $\chi^2(44) = 65.215$, $p = .020$). Analyses of missing data patterns revealed that missing data were unrelated to the dependent variables. To check the robustness of running analyses using listwise deletion, we also ran pooled analyses using ten imputed datasets. The differences in included cases was relatively small (alcohol use: 13.716 vs. 13.404; binge drinking: 10.990 vs. 10.764; tobacco use: 2.736 vs. 2.653; cannabis use: 1.849 vs. 1.804). Since the results were nearly identical to one another, we included the results found using listwise deletion. The pooled regression results are available upon reasonable request.

Light and heavy users

Since the original analyses did not take into account the intensity of use, we ran separate analyses for light and heavy users to assess the robustness of the original analyses. Due to low response counts (<10 respondents) we produced a number of unreliable estimates. These estimates are listed below and should be ignored when examining the results of these particular analyses. Differences between the main analyses and the control analyses were reported in the article, if relevant. The control analyses can be found in Additional File 4.

Alcohol use—Light drinkers (n=11,508) were (a) students drinking one to five glasses of alcohol per week before and during the pandemic, and (b) students drinking more than five glasses per week before the pandemic and one to five glasses per week during the pandemic. Heavy drinkers (n=1,896) were (a) students drinking more than five glasses of alcohol per week before and during the pandemic, and (b) students drinking one to five glasses per week before the pandemic and more than five glasses per week during the pandemic [1]. Biased estimates among heavy drinkers were produced for health condition and the dummy for moved away from parental home.

Binge drinking—Light bingers (n=9,826) were (a) students who binge drank less than once a week before and during the pandemic, and (b) students who binge drank at least weekly before the pandemic and less than once a week during the pandemic. Heavy bingers (n=938) were (a) students who binge drank at least weekly before and during the pandemic, and (b) students who binge drank less than once a week before the pandemic, and at least weekly during the pandemic. For light and heavy bingers, unreliable estimates were produced for the dummy for moved away from parental home. For light bingers, we also produced biased estimates for the dummy for increased financial situation.

Tobacco use—Low-rate daily smokers (n=2,038) were (a) students who smoked one to five cigarettes per day before and during the pandemic, and (b) students who smoked more than five cigarettes per day before the pandemic and one to five cigarettes during the pandemic. High-rate daily smokers (n=615) were (a) students who smoked more than five cigarettes per day before and during the pandemic, and (b) students who smoked one to five cigarettes per day before the pandemic and more than five cigarettes during the pandemic [2,3]. For high-rate daily smokers, we produced unreliable estimates for the dummy for moved away from parental home and the dummy for increased financial situation.

Cannabis use—Light cannabis users (n=1,212) were (a) students who used cannabis less than once a week before and during the pandemic, and (b) students who used cannabis at least weekly before the pandemic and less than once a week during the pandemic.

Heavy cannabis users (n=592) were (a) students who used cannabis at least weekly before and during the pandemic, and (b) students who used cannabis less than once a week before the pandemic, and at least weekly during the pandemic. Among light and heavy cannabis users, biased estimates were produced for the dummy for moved away from parental home, the dummy for increased financial situation, and health condition. Among heavy cannabis users, we also produced biased estimates for parental educational attainment and COVID-19 diagnosis.

References

1. Wood, A.M.; Kaptoge, S.; Butterworth, A.S.; Willeit, P.; Warnakula, S.; Bolton, T.; Paige, E.; Paul, D.S.; Sweeting, M.; Burgess, S. Risk thresholds for alcohol consumption: combined analysis of individual-participant data for 599 912 current drinkers in 83 prospective studies. *The Lancet* **2018**, *391*, 1513-1523.
2. Schane, R.E.; Ling, P.M.; Glantz, S.A. Health effects of light and intermittent smoking: a review. *Circulation* **2010**, *121*, 1518-1522, doi:10.1161/CIRCULATIONAHA.109.904235.
3. Robertson, L.; Iosua, E.; McGee, R.; Hancox, R.J. Nondaily, Low-Rate Daily, and High-Rate Daily Smoking in Young Adults: A 17-Year Follow-Up. *Nicotine Tob Res* **2016**, *18*, 943-949, doi:10.1093/ntr/ntv167.
