# Supplementary material for: Substance Use among Belgian Higher Education Students before and during the First Wave of the COVID-19 Pandemic
Source: Int J Environ Res Public Health. 2022 Apr 5;19(7):4348. doi: 10.3390/ijerph19074348 (PMC8998911; doi:10.3390/ijerph19074348)
Supplement: Supplementary file 1 [file ijerph-19-04348-s001.zip › Supplementary_3_assumption_testing.pdf]

International Journal of
Environmental Research and Public Health

MDPI

Supplementary 3

Assumption testing

Proportional odds assumption

Since we could argue that our dependent variables are ordinal, we checked whether we could perform ordered logistic regression by testing the proportional odds assumption using tests of parallel lines. We found that our data violate this assumption (alcohol use: $\chi^2(17) = 151.877$, $p < .001$; binge drinking: $\chi^2(17) = 66.758$, $p < .001$; tobacco use: $\chi^2(17) = 164.376$, $p < .001$; cannabis use: $\chi^2(17) = 40.808$, $p < .001$). Multinomial logistic regression analyses are, therefore, preferred.

Assumptions of multinomial logistic regression analyses

Multicollinearity—We estimated correlations between the independent variables and entered the independent variables into linear regression analyses to check Variance Inflation Factors (VIFs). No correlations higher than 0.59 or lower than -0.37 and VIFs higher than 1.65 were found, confirming the assumption of no multicollinearity.

Outliers or highly influential cases—We checked scatterplots, Cook's distance scores, standardized residuals, centered leverage values and DFBETA scores. We found no indications that atypical response patterns were influential.

Linearity—We checked whether all independent continuous variables had a linear relationship with the logit of the dependent variable using Box-Tidwell tests. Bonferroni corrections were applied to compensate for simultaneously running multiple tests of independence. For binge drinking, social activity showed a non-linear relationship. We made a distinction between (1) median; (2) below median; and (3) above median.
