# Supplementary material for: Substance Use among Belgian Higher Education Students before and during the First Wave of the COVID-19 Pandemic
Source: Int J Environ Res Public Health. 2022 Apr 5;19(7):4348. doi: 10.3390/ijerph19074348 (PMC8998911; doi:10.3390/ijerph19074348)
Supplement: Supplementary file 1 [file ijerph-19-04348-s001.zip › Supplementary_4_control_analyses.pdf]

International Journal of
Environmental Research and Public Health

MDPI

Supplementary 4

Control analyses

Table S5. Multinomial regression analyses for alcohol use: light drinkers (n=11,508) and heavy drinkers (n=1,896)

		Light drinkers				Heavy drinkers			
		Increase vs. No change		Decrease vs. No change		Increase vs. No change		Decrease vs. No change	
		AO R	95% CI	AOR	95% CI	AOR	95% CI	AOR	95% CI
Sex	Male	1.00		1.00		1.00		1.00	
	Female	1.01	0.84-1.20	1.01	0.84-1.20	1.01	0.84-1.20	1.01	0.84-1.20
Age		0.99	0.94-1.04	0.83***	0.79-0.86	0.92	0.82-1.03	0.77***	0.69-0.87
Parent(s) HE degree	No	1.00		1.00		1.00		1.00	
	Yes	0.99	0.84-1.16	1.03	0.90-1.18	0.86	0.57-1.31	0.85	0.55-1.33
Study year	First-year	1.00		1.00		1.00		1.00	
	Higher year	1.05	0.85-1.30	1.08	0.91-1.29	0.77	0.43-1.35	1.08	0.60-1.96
Institution type	College of applied sciences	1.00		1.00		1.00		1.00	
	University	0.88	0.76-1.01	1.33***	1.18-1.50	0.88	0.62-1.25	1.17	0.81-1.69
Health condition	No	1.00		1.00		1.00		1.00	
	Underlying condition	1.17	0.94-1.47	1.05	0.86-1.27	*2.85***	*1.42-5.73*	*2.22**	*1.07-4.61*
COVID-19 diagnosis	No	1.00		1.00		1.00		1.00	
	(Un)confirmed probably or yes	0.95	0.76-1.18	1.05	0.88-1.26	0.78	0.50-1.21	0.82	0.52-1.31
Living situation	Parental home	1.00		1.00		1.00		1.00	
	Moved back to parental home	0.90	0.77-1.06	1.89***	1.66-2.15	0.71	0.49-1.04	1.34	0.91-1.98
	Outside of parental home	0.86	0.69-1.07	1.06	0.88-1.27	0.80	0.52-1.22	0.84	0.54-1.33
	Moved away from parental home	1.21	0.79-1.85	0.95	0.65-1.40	*3.43**	*1.04-11.33*	*1.92*	*0.54-6.83*
Financial situation	No change	1.00		1.00		1.00		1.00	
	Decrease	1.20*	1.03-1.41	1.11	0.98-1.27	1.30	0.90-1.89	1.06	0.71-1.57
	Increase	1.22	0.87-1.72	1.56**	1.18-2.06	0.70	0.39-1.28	0.81	0.44-1.50
Social activity		0.98	0.94-1.02	1.08***	1.04-1.12	0.93	0.84-1.02	1.06	0.96-1.16
Perceived threat		1.02	0.98-1.07	0.97	0.94-1.01	0.99	0.90-1.10	0.89*	0.80-0.99
Academic stress		1.05	0.95-1.16	0.92	0.85-1.00	1.04	0.83-1.30	0.86	0.68-1.08
Academic dissatisfaction		0.99	0.89-1.09	1.12*	1.02-1.22	1.04	0.82-1.31	1.06	0.82-1.36
Depressive symptoms		1.05***	1.03-1.07	1.04***	1.03-1.06	1.07***	1.03-1.11	1.03	0.99-1.07

*** p < .001; ** p < .01; * p < .05

Results in italics are biased estimates and should be ignored.

Table S6. Multinomial regression analyses for binge drinking: light bingers (n=9,826) and heavy bingers (n=938)

		**Light bingers**				**Heavy bingers**			
		**Increase vs. No change**		**Decrease vs. No change**		**Increase vs. No change**		**Decrease vs. No change**	
		**AOR**	**95% CI**	**AOR**	**95% CI**	**AOR**	**95% CI**	**AOR**	**95% CI**
Sex	Male	1.00		1.00		1.00		1.00	
	Female	0.70	0.43-1.12	0.83	0.66-1.04	1.50*	1.06-2.13	0.66*	0.44-0.99
Age		1.06	0.91-1.22	0.82***	0.77-0.88	1.13*	1.01-1.27	0.91	0.79-1.05
Parent(s) HE degree	No	1.00		1.00		1.00		1.00	
	Yes	1.42	0.89-2.27	1.22	0.99-1.51	0.84	0.56-1.26	0.81	0.49-1.32
Study year	First-year	1.00		1.00		1.00		1.00	
	Higher year	0.91	0.50-1.67	1.33	1.00-1.76	0.78	0.48-1.26	1.83*	1.01-3.31
Institution type	College of applied sciences	1.00		1.00		1.00		1.00	
	University	0.72	0.47-1.09	1.36**	1.12-1.66	0.87	0.61-1.23	1.03	0.68-1.55
Health condition	No	1.00		1.00		1.00		1.00	
	Underlying condition	1.11	0.62-1.99	0.84	0.63-1.12	1.03	0.63-1.67	0.83	0.45-1.54
COVID-19 diagnosis	No	1.00		1.00		1.00		1.00	
	(Un)confirmed probably or yes	0.89	0.47-1.67	1.12	0.84-1.50	0.95	0.59-1.52	0.84	0.47-1.49
Living situation	Parental home	1.00		1.00		1.00		1.00	
	Moved back to parental home	0.99	0.59-1.65	1.69***	1.35-2.12	0.74	0.49-1.10	1.21	0.77-1.90
	Outside of parental home	1.07	0.62-1.86	0.66**	0.51-0.86	1.22	0.79-1.90	1.11	0.66-1.89
	Moved away from parental home	*1.83*	*0.75-4.43*	0.63	0.38-1.04	*2.18*	*0.88-5.42*	*0.37*	*0.07-1.84*
Financial situation	No change	1.00		1.00		1.00		1.00	
	Decrease	1.36	0.89-2.09	0.75**	0.61-0.92	1.14	0.79-1.62	0.78	0.50-1.22
	Increase	*1.41*	*0.57-3.45*	1.12	0.73-1.73	1.67	0.82-3.36	1.07	0.49-2.36
Social activity	Median	1.00		1.00		1.00		1.00	
	Below median	1.97*	1.11-3.50	1.18	0.94-1.49	0.78	0.49-1.22	0.55*	0.31-0.95
	Above median	1.80	0.99-3.29	1.10	0.86-1.39	0.56*	0.36-0.89	0.96	0.57-1.60
Perceived threat		1.00	0.88-1.13	0.96	0.90-1.02	1.13*	1.03-1.24	1.04	0.93-1.16
Academic stress		1.19	0.87-1.64	0.92	0.80-1.05	1.29*	1.01-1.66	0.92	0.70-1.22
Academic dissatisfaction		1.09	0.82-1.46	1.07	0.93-1.23	0.82	0.64-1.06	1.08	0.81-1.44
Depressive symptoms		1.07**	1.02-1.12	1.01	0.99-1.03	1.09***	1.05-1.13	1.03	0.99-1.07

*** p < .001; ** p < .01; * p < .05

Results in italics are biased estimates and should be ignored.

Table S7. Multinomial regression analyses for tobacco use: low-rate daily smokers (n=2,038) and high-rate daily smokers (n=615)

		**Low-rate daily smokers**				**High-rate daily smokers**			
		**Increase vs. No change**		**Decrease vs. No change**		**Increase vs. No change**		**Decrease vs. No change**	
		**AOR**	**95% CI**	**AOR**	**95% CI**	**AOR**	**95% CI**	**AOR**	**95% CI**
Sex	Male	1.00		1.00		1.00		1.00	
	Female	0.69*	0.51-0.93	1.08	0.86-1.34	1.96**	1.23-3.12	1.32	0.66-2.62
Age		1.10*	1.00-1.22	1.13***	1.05-1.21	0.89	0.77-1.03	0.90	0.73-1.13
Parent(s) HE degree	No	1.00		1.00		1.00		1.00	
	Yes	0.62**	0.44-0.87	0.95	0.73-1.23	1.00	0.63-1.58	1.64	0.79-3.42
Study year	First-year	1.00		1.00		1.00		1.00	
	Higher year	0.78	0.52-1.15	0.86	0.65-1.13	0.58	0.29-1.15	0.73	0.27-1.99
Institution type	College of applied sciences	0.00		1.00		1.00		1.00	
	University	0.67**	0.50-0.90	0.61***	0.50-0.76	0.97	0.62-1.52	0.77	0.40-1.51
Health condition	No	1.00		1.00		1.00		1.00	
	Underlying condition	1.16	0.75-1.80	0.90	0.64-1.26	1.04	0.58-1.86	1.16	0.50-2.70
COVID-19 diagnosis	No	1.00		1.00		1.00		1.00	
	(Un)confirmed probably or yes	1.59*	1.09-2.32	1.14	0.85-1.53	1.00	0.54-1.82	1.08	0.46-2.58
Living situation	Parental home	1.00		1.00		1.00		1.00	
	Moved back to parental home	0.59**	0.41-0.83	1.06	0.85-1.32	0.69	0.40-1.21	1.13	0.51-2.53
	Outside of parental home	2.39***	1.65-3.46	0.72*	0.53-0.98	0.91	0.53-1.55	1.05	0.45-2.42
	Moved away from parental home	1.43	0.65-3.15	0.75	0.40-1.40	*0.45*	*0.17-1.18*	*1.39*	*0.39-4.89*
Financial situation	No change	1.00		1.00		1.00		1.00	
	Decrease	1.30	0.96-1.77	1.65***	1.32-2.06	1.22	0.78-1.90	1.07	0.54-2.13
	Increase	0.83	0.45-1.52	2.01***	1.39-2.91	*1.64*	*0.62-4.33*	*4.03*	*1.28-12.65*
Social activity		1.05	0.96-1.14	1.00	0.94-1.06	0.96	0.85-1.08	0.99	0.82-1.19
Perceived threat		1.07	0.99-1.16	1.08**	1.02-1.15	1.03	0.91-1.17	1.01	0.83-1.22
Academic stress		1.41**	1.14-1.74	1.10	0.96-1.27	1.03	0.77-1.38	0.97	0.63-1.49
Academic dissatisfaction		0.82	0.67-1.01	0.98	0.84-1.13	1.18	0.87-1.58	1.33	0.86-2.05
Depressive symptoms		1.05**	1.02-1.09	1.00	0.98-1.03	1.05*	1.01-1.10	0.99	0.93-1.06

*** p < .001; ** p < .01; * p < .05

Results in italics are biased estimates and should be ignored.

Table S8. Multinomial regression analyses for cannabis use: light cannabis users (n=1,212) and heavy cannabis users (n=592)

		**Light cannabis users**				**Heavy cannabis users**			
		**Increase vs. No change**		**Decrease vs. No change**		**Increase vs. No change**		**Decrease vs. No change**	
		**AOR**	**95% CI**	**AOR**	**95% CI**	**AOR**	**95% CI**	**AOR**	**95% CI**
Sex	Male	1.00		1.00		1.00		1.00	
	Female	1.03	0.52-2.05	0.54*	0.34-0.87	1.63*	1.10-2.40	0.60	0.29-1.25
Age		0.98	0.80-1.20	0.89	0.77-1.03	1.02	0.90-1.16	0.89	0.70-1.13
Parent(s) HE degree	No	1.00		1.00		1.00		1.00	
	Yes	0.77	0.37-1.61	0.56*	0.33-0.96	0.91	0.59-1.39	*1.92*	*0.74-4.99*
Study year	First-year	1.00		1.00		1.00		1.00	
	Higher year	1.29	0.55-3.06	1.30	0.71-2.36	1.01	0.60-1.70	1.17	0.47-2.89
Institution type	College of applied sciences	1.00		1.00		1.00		1.00	
	University	1.02	0.57-1.85	1.60	1.04-2.44	1.22	0.84-1.77	1.41	0.71-2.80
Health condition	No	1.00		1.00		1.00		1.00	
	Underlying condition	0.81	0.30-2.15	1.17	0.59-2.31	0.93	0.52-1.66	*1.34*	*0.49-3.69*
COVID-19 diagnosis	No	1.00		1.00		1.00		1.00	
	(Un)confirmed probably or yes	*1.05*	*0.49-2.27*	0.98	0.56-1.73	1.29	0.77-2.16	*1.41*	*0.60-3.36*
Living situation	Parental home	1.00		1.00		1.00		1.00	
	Moved back to parental home	1.00	0.47-2.10	2.17**	1.28-3.68	1.06	0.65-1.75	2.43*	1.04-5.70
	Outside of parental home	0.64	0.31-1.32	0.37***	0.22-0.62	1.32	0.84-2.09	1.86	0.76-4.52
	Moved away from parental home	*0.37*	*0.07-1.98*	*0.23***	*0.08-0.66*	*1.79*	*0.74-4.35*	*0.86*	*0.10-7.75*
Financial situation	No change	1.00		1.00		1.00		1.00	
	Decrease	1.48	0.80-2.74	1.04	0.66-1.64	1.05	0.71-1.56	1.02	0.50-2.11
	Increase	*0.98*	*0.30-3.24*	*1.46*	*0.66-3.23*	0.30**	0.14-0.63	*0.56*	*0.15-2.09*
Social activity		0.96	0.80-1.15	1.01	0.89-1.15	1.08	0.97-1.20	1.02	0.84-1.23
Perceived threat		1.10	0.92-1.31	0.88*	0.78-1.00	1.03	0.92-1.14	0.99	0.82-1.19
Academic stress		0.77	0.51-1.16	0.80	0.60-1.09	1.08	0.83-1.42	1.40	0.82-2.37
Academic dissatisfaction		0.99	0.65-1.50	0.93	0.69-1.25	1.12	0.86-1.47	1.03	0.64-1.68
Depressive symptoms		1.03	0.96-1.10	1.04	0.99-1.09	1.06**	1.02-1.11	1.03	0.96-1.11

*** p < .001; ** p < .01; * p < .05

Results in italics are biased estimates and should be ignored.
